# Supplementary material for: Elucidating the Link: Chronic Obstructive Pulmonary Disease and the Complex Interplay of Gastroesophageal Reflux Disease and Reflux-Related Complications
Source: Medicina (Kaunas). 2023 Jul 8;59(7):1270. doi: 10.3390/medicina59071270 (PMC10384576; doi:10.3390/medicina59071270)
Supplement: Supplementary file 1 [file medicina-59-01270-s001.zip › medicina-2423164-supplementary.pdf]

Supplement Table S1. The prevalence for GERD and GERD-associated complications in patients with COPD based on age and gender. OR, odds ratio; GERD, gastroesophageal reflux, disease; COPD, chronic obstructive pulmonary disease; w/, with; w/o, without.

		case	prevalence (%)	p value
		Nonerosive reflux disease		
AGE<=54	COPD	31,875	26.30%	<0.01
	Non-COPD	249,202	7.40%	
55<=AGE<=74	COPD	143,898	27.90%	<0.01
	Non-COPD	362,287	21.90%	
AGE>=75	COPD	100,507	28.30%	<0.01
	Non-COPD	257,255	22.90%	
Male	COPD	116,642	24.70%	<0.01
	Non-COPD	357,010	13.60%	
Female	COPD	159,634	30.80%	<0.01
	Non-COPD	511,719	14.60%	
		erosive esophagitis		
	COPD	1,085	1.20%	<0.01
AGE<=54	Non-COPD	10,153	0.30%	
	COPD	3,866	1.00%	<0.01
55<=AGE<=74	Non-COPD	11,364	0.90%	
	COPD	2,424	0.90%	<0.01
AGE>=75	Non-COPD	6,844	0.80%	
	COPD	3,442	1.00%	<0.01
Male	Non-COPD	13,581	0.60%	
	COPD	3,906	1.10%	<0.01
Female	Non-COPD	14,780	0.50%	
		Esophageal stricture		
AGE<=54	COPD	127	14.7	<0.01
	Non-COPD	843	2.7	
55<=AGE<=74	COPD	798	21.3	<0.01
	Non-COPD	1,952	15	
AGE>=75	COPD	857	33.4	<0.01
	Non-COPD	2,304	26.5	
Male	COPD	756	21.3	<0.01
	Non-COPD	2,143	9.4	
Female	COPD	1,017	28.1	<0.01
	Non-COPD	2,956	9.8	
		Barrett's Esophagus w/o dysplasia		
AGE<=54	COPD	585	0.70%	<0.01
	Non-COPD	3,370	0.10%	
55<=AGE<=74	COPD	3,352	0.90%	<0.01

	Non-COPD	8,831	0.70%	
AGE>=75	COPD	2,144	0.80%	<0.01
	Non-COPD	5,424	0.60%	
Male	COPD	3,571	1.00%	<0.01
	Non-COPD	10,567	0.50%	
Female	COPD	2,509	0.70%	<0.01
	Non-COPD	7,058	0.20%	
		Barrett's Esophagus w/ dysplasia		
AGE<=54	COPD	13	1.4	<0.01
	Non-COPD	53	0.1	
55<=AGE<=74	COPD	67	1.8	>0.05
	Non-COPD	239	1.9	
AGE>=75	COPD	48	1.9	>0.05
	Non-COPD	112	1.3	
Male	COPD	97	2.7	<0.01
	Non-COPD	276	0.9	
Female	COPD	31	0.8	<0.01
	Non-COPD	128	0.4	
		Esophageal cancer		
AGE<=54	COPD	29	3.2	<0.01
	Non-COPD	251	0.8	
55<=AGE<=74	COPD	399	10.7	<0.01
	Non-COPD	1,165	8.9	
AGE>=75	COPD	117	4.5	<0.01
	Non-COPD	436	5	
Male	COPD	453	12.6	<0.01
	Non-COPD	1,450	6.3	
Female	COPD	152	4.2	<0.01
	Non-COPD	402	1.3	

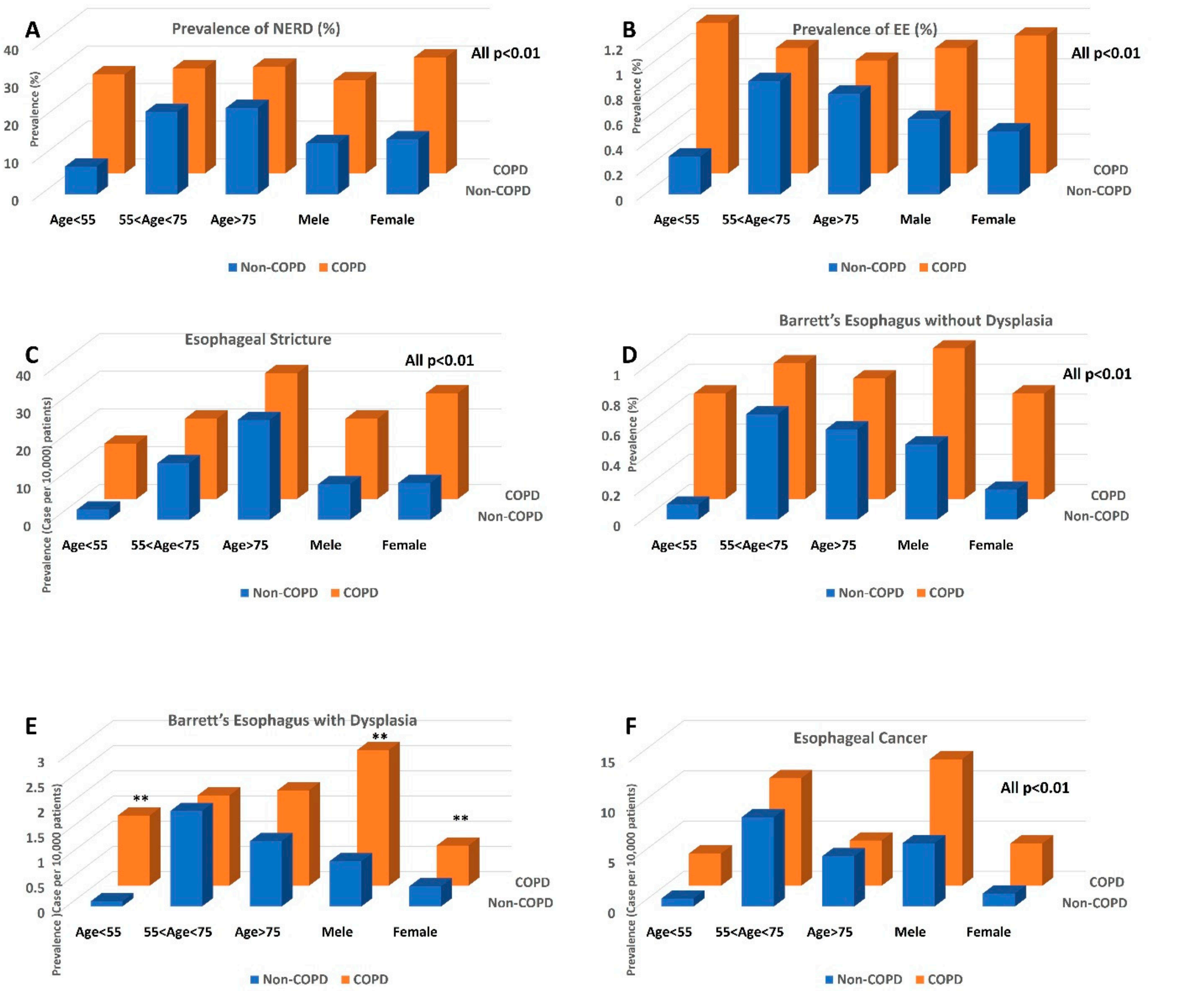

Supplement Figure S1. Bar graph of prevalence for COPD patients with GERD or GERD-related complications based on age or gender. A, Prevalence of NERD with or without COPD in different age groups and gender. B, Prevalence of EE in patients with or without COPD in different age groups and gender. C, Prevalence of Esophageal stricture with or without COPD in different age groups and gender. D, Prevalence of Barrett's Esophagus without dysplasia in patients with or without COPD in different age groups and gender. E, Prevalence of Barrett's Esophagus with dysplasia in patients with or without COPD in different age groups and gender. F, Prevalence of Esophageal cancer in patients with or without COPD in different age groups and gender. NERD, non-erosive reflux disease; EE, erosive esophagitis; COPD, chronic obstructive pulmonary disease. ** p<0.01.
